# Iron Absorption: Factors, Limitations, and Improvement Methods

**DOI:** 10.1021/acsomega.2c01833

**Published:** 2022-06-10

**Authors:** Elif Piskin, Danila Cianciosi, Sukru Gulec, Merve Tomas, Esra Capanoglu

**Affiliations:** † Faculty of Engineering and Natural Sciences, Food Engineering Department, Istanbul Sabahattin Zaim University, Halkali, 34303 Istanbul, Turkey; ‡ Faculty of Medicine, Department of Clinical Sciences, Polytechnic University of Marche, via Pietro Ranieri, 60131 Ancona, Italy; § Molecular Nutrition and Human Physiology Laboratory, Department of Food Engineering, İzmir Institute of Technology, 35430 Urla, İzmir; ∥ Department of Food Engineering, Faculty of Chemical and Metallurgical Engineering, Istanbul Technical University, Maslak, 34469 Istanbul, Turkey

## Abstract

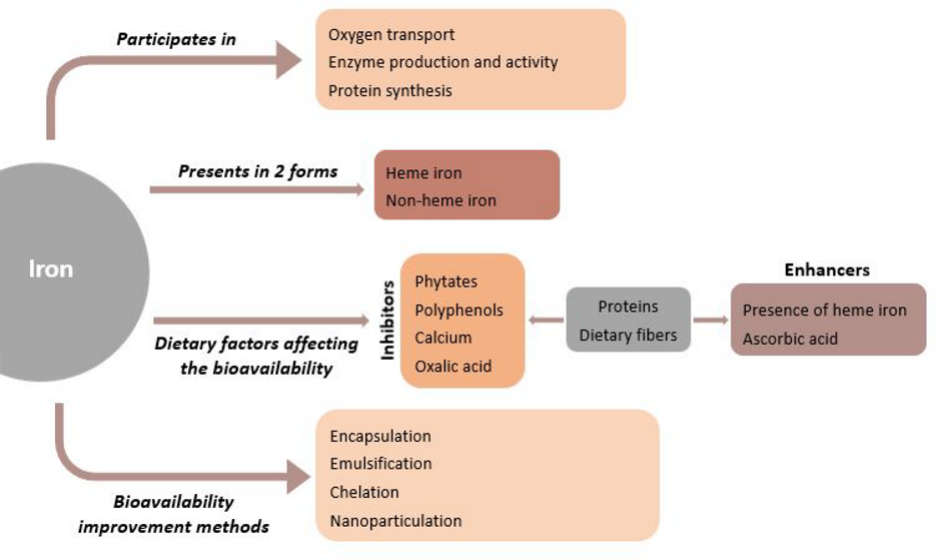

Iron is an essential element for human life since it participates in many functions in the human body, including oxygen transport, immunity, cell division and differentiation, and energy metabolism. Iron homeostasis is mainly controlled by intestinal absorption because iron does not have active excretory mechanisms for humans. Thus, efficient intestinal iron bioavailability is essential to reduce the risk of iron deficiency anemia. There are two forms of iron, heme and nonheme, found in foods. The average daily dietary iron intake is 10 to 15 mg in humans since only 1 to 2 mg is absorbed through the intestinal system. Nutrient–nutrient interactions may play a role in dietary intestinal iron absorption. Dietary inhibitors such as calcium, phytates, polyphenols and enhancers such as ascorbic acid and proteins mainly influence iron bioavailability. Numerous studies have been carried out for years to enhance iron bioavailability and combat iron deficiency. In addition to traditional methods, innovative techniques are being developed day by day to enhance iron bioavailability. This review will provide information about iron bioavailability, factors affecting absorption, iron deficiency, and recent studies on improving iron bioavailability.

## 1 Introduction

Iron is one of the essential heavy metals for human nutrition, and it is a vital element for human life.^1^ It plays critical roles in oxygen and electron transport, cell division, differentiation, and regulation of gene expression.^2^ 70% of the iron in the human body binds to the hemoglobin, the pigment of red blood cells (RBCs) that gives the blood its red color, and the rest binds to other proteins, such as myoglobin, transferrin, and ferritin, or is stored in the cells. The reticuloendothelial system, which clears damaged RBCs by macrophages of the spleen, liver, and bone marrow, plays a role in systemic iron homeostasis.^3^

Two types of iron can be found in foods, including heme and nonheme. Heme iron is present only in animal products such as meat, fish, and poultry, whereas nonheme iron is found in fruits, vegetables, dried beans, nuts, grain products, and meat.^4^ Heme iron is absorbed with better efficiency from the intestine than nonheme iron.^5^ Tight control of dietary iron absorption is essential to maintain an iron level within the normal range to reduce the risk of iron deficiency.

The standard definition of intestinal nutrient bioavailability is the portion of the absorbed and utilized nutrients from digested foods through enterocyte cells of the intestine. The absorption rate of iron has been reported as 25–30% in the consumption of organ meats, 7–9% in green leafy vegetables, 4% in grains, and 2% in dried legumes, indicating that food types or other dietary factors might influence iron bioavailability.^6^ For instance, ascorbic acid is a well-known dietary factor improving iron bioavailability;^7^ however, calcium, polyphenols, and phytates reduce intestinal iron absorption.^8,9^ Thus, we need to consider the type of foods in our diet to maintain an iron balance in the body. Inadequate iron absorption leads to iron deficiency anemia. Iron deficiency is the most common nutritional deficiency worldwide. It negatively affects the cognitive development in infants, children, and adolescents. Maternal iron deficiency anemia may cause low birth weight and preterm delivery.^10^ The report from the World Health Organization indicated that more than 27% of the world’s population experiences iron deficiency anemia. Therefore, the prevention of iron deficiency is crucial for these groups.

In this review, iron bioavailability, iron deficiency and anemia, dietary factors affecting iron bioavailability, and recent studies on increasing iron bioavailability were determined using the search terms “iron, iron bioavailability, iron absorption, iron deficiency and anemia, iron bioavailability enhancers/inhibitors, iron bioavailability improvement, encapsulation of iron, and nanoparticulation of iron” via PubMed, Web of Science, and Google Scholar databases. While papers and reports having a scientific quality are included in the study, articles written in languages other than English and the ones for which full text could not be reached formed the exclusion criteria of the study. In addition, under the title “iron bioavailability improvement methods” only the studies published after the year 2014 are included. As a result of the search, a total of 1254 studies were found, and the article was created by referring to 178 sources at the end of the eliminations made in line with the inclusion/exclusion criteria.

## 2 Iron Absorption

Dietary iron absorption is primarily performed through enterocyte cells on the duodenum and upper jejunum of the small intestine. Because humans do not have an active iron excretion system, intestinal iron absorption is critical for maintaining iron balance in the body.^11^ A typical Western diet contains 7 mg of iron per 1000 kcal; however, only 1–2 mg is absorbed daily.^12^ Nonheme iron accounts for up to 90% of the iron consumed through food. It is in the form of Fe^+3^ complexes in foods, and its absorption is affected by dietary factors and iron status in the human body. Contrary to nonheme iron, heme iron has a high absorption rate and is less affected by dietary factors. Heme iron accounts for 10% of dietary iron.^13,14^ Two different molecular mechanisms are involved in absorbing heme and nonheme iron in the intestines; however, iron enters the same intracellular pool as newly absorbed heme or nonheme iron and can be stored in iron storage protein.^11^ Body iron status and dietary food types may influence intestinal iron absorption. Furthermore, cast iron pots and cookware can also be a source of significant quantities of dietary iron and, most importantly, the form of iron in food as heme or nonheme. In addition, the diet consumption patterns of nonheme iron absorption are affected by the diet.^15^ Getting enough iron is one of the requirements for a healthy life.^16^ Reference Dietary (Daily) Allowance (RDA) values, the average daily nutrient intake level for healthy people to meet the adequate nutrient needs of the body, have been established by the Food and Nutrition Board, Institute of Medicine (IOM).^17,18^ In addition, for infants from birth to 6 months, an average intake (AI) for iron equivalent to the mean intake of iron in healthy, breastfed infants are established. According to the IOM, the AI value for infants aged 0–6 months and the RDA value for those aged 7–13 months are 0.27 mg/day and 11 mg/day, respectively. For children aged 1–3 years, 4–8 years, and 9–13 years; these values are 7 mg/day, 10 mg/day, and 8 mg/day, respectively. In adolescence (14–18 years), the RDA value changes between men and females. While the RDA for male adolescents is 11 mg/day, the RDA value is 15 mg/day for female adolescents. For adults over 19, the RDA value is 8 mg/day and 18 mg/day for males and females, respectively. In addition, the RDA value for lactating women younger than 18 years old is 10 mg/day, and it is 9 mg/day for those over 18 years of age. Furthermore, RDA changes for vegetarians. The RDA for a vegetarian man is 14 mg/day, and for vegetarian females aged between 14 and 18 years, 19 and 50 years, and over 51 years, it is 26 mg/day, 14 mg/day, and 33 mg/day, respectively. Iron deficiency occurs when the body’s iron requirement is unmet, which may cause health problems.^18^

## 3 Iron Deficiency and Anemia (IDA)

Iron deficiency and iron deficiency anemia are global health problems and common medical conditions seen in daily clinical practice.^19^ Iron deficiency is when the amount of iron needed by the body cannot be met due to some physiological consequences, including blood loss and limited dietary supply.^19,20^ The World Health Organization (WHO) defines iron deficiency anemia as a hemoglobin (Hb) level of less than 13 g/dl in men and 12 g/dl in women.^21^ Iron deficiency is the most common nutritional deficiency worldwide. It is a significant health problem for children, adolescents, pregnant women, and those with low socioeconomic status, especially in developing countries. Furthermore, IDA also affects many people around the industrialized world.^22^ However, the prevalence of iron deficiency in developing countries is approximately four times higher than in developed countries.^23^ Recently, up to one-third of the world’s population is suffering from iron deficiency. Infants, the elderly, and women, especially during menstruation and pregnancy, are at high risk for IDA.^24^

**Figure 1:**
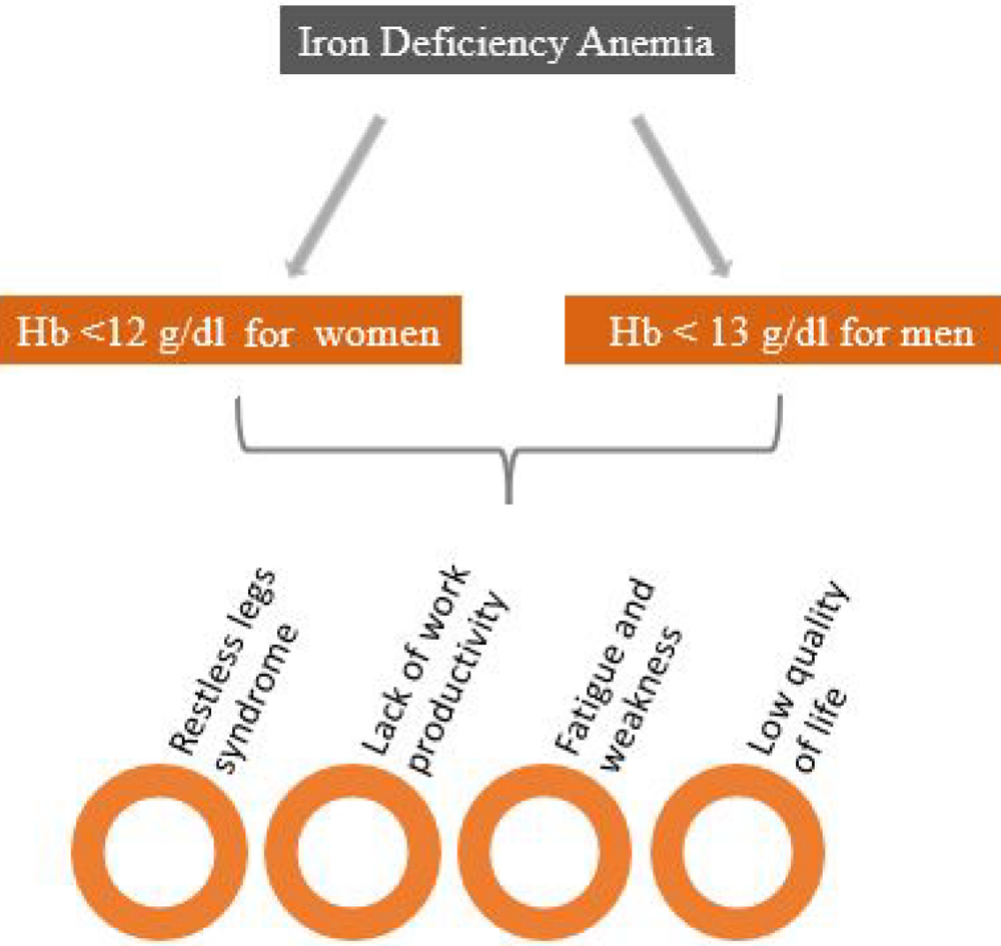
Iron deficiency anemia causes and symptoms.

IDA is a severe problem that negatively affects growth, cognitive function, and behaviors in infants.^25−28^ It induces some health problems in adults, including restless legs syndrome (RLS),^29^ diminished quality of life,^30^ fatigue and weakness,^31^ trouble concentrating, and lack of work productivity.^32^ In pregnant women, low iron levels may affect the birth weight and cause prematurity^33,34^ and even mortality in the mother and child.^35^ In addition, a link between preoperative anemia and increased risk of 30-day morbidity and mortality in patients undergoing major noncardiac surgery was demonstrated.^36^ A schematic summary of the cause and symptoms of iron deficiency anemia is provided in Figure 1.

## 4 Bioavailability of Iron from Dietary Sources

Heme iron contributes 10–15% of the total iron intake. However, since heme iron is absorbed better than nonheme iron, with an approximate 15–35% of absorption, it can account for more than 40% of total intestinal iron absorption.^37^ In contrast to heme iron, nonheme iron is found in animal and plant sources (i.e., cereals, beans, and herbs) and iron-enriched or fortified foods such as iron-fortified cereal.^38,39^

The iron content of foods does not indicate its bioavailability because iron absorption depends on some factors, mainly the form of iron. Because plants mainly contain nonheme iron, even if its iron content is high, absorption of iron is low due to plant-based molecule–iron interactions.^40^ Red meat is the most significant source of iron since it is rich in heme iron, which is highly bioavailable.^41,42^ Since 30–70% of the iron in meat is in the form of heme, the iron requirement for humans is mainly met by red meat in developed countries. In contrast, in underdeveloped and developing countries, iron intake depends on plant-based diets that contain mostly nonheme iron, often absorbed less than 10%.^42,43^ Therefore, the consumption of iron-containing food is one of the main factors determining the body iron status.

## 5 Dietary Factors Affecting Iron Bioavailability

**Figure 2:**
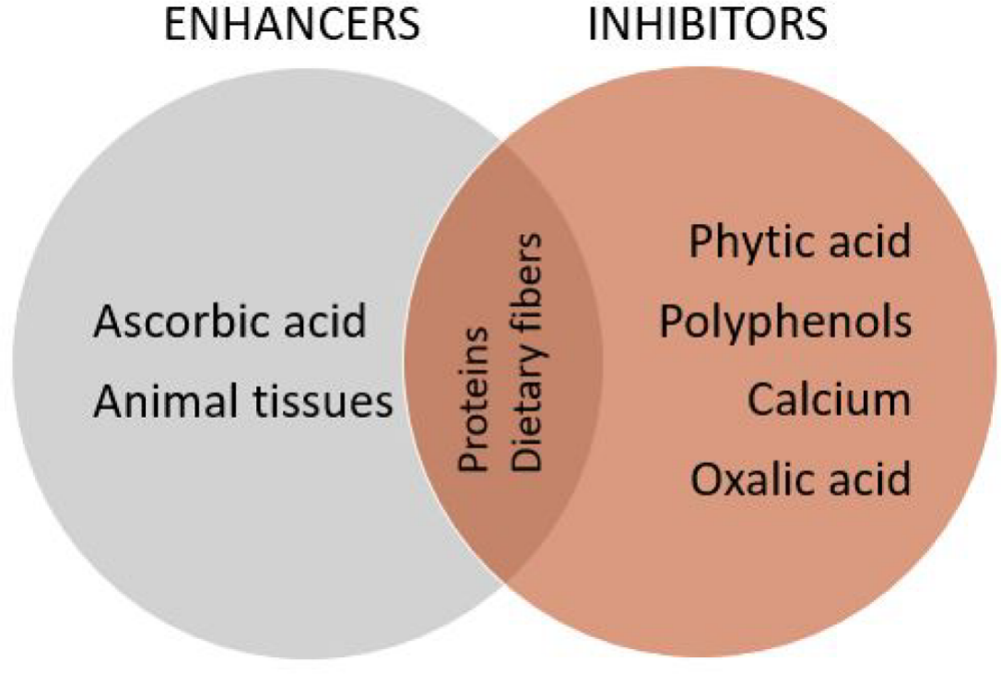
Main enhancers and inhibitors of iron bioavailability.

The absorption of iron heavily depends on the physical state of iron as ferrous and ferric.^44^ Nonheme iron in the diet is primarily in an oxidized or ferric form, although ferrous iron is more likely to be carried into enterocytes.^11^ Ferric iron is precipitated in solutions with a pH higher than 3, whereas most ferrous iron remains soluble at a neutral pH. Therefore, ferric iron must first be solubilized and chelated in the stomach to be absorbed in the less acidic proximal small intestine.^45^ The chelation happens rapidly by the other components in food as iron is released in the intestinal lumen. These chelators may be enhancers and inhibitors influencing iron absorption via iron solubility.^6^ Therefore, diet composition is one of the main factors influencing the absorption of nonheme iron.^46^Figure 2 shows the main enhancers and inhibitors of iron bioavailability, and Table 1 shows the relevant studies.

**Table 1:** Main Enhancers and Inhibitors of Iron Absorption

type of study	component/dose	experimental model	main results	reference
*in vivo*	ascorbic acid/25 to 1000 mg	63 male subjects	As vitamin C dose was increased from 25 to 1000 mg, absorption of iron showed an increase from 0.8% to 7.1% in male subjects who were fed with a liquid formula meal containing 4.1 mg of iron.	(56)
*in vitro*	ascorbic acid/-	caco-2 cell	Ascorbic acid enhanced the absorption only when it was added along with the digests to Caco-2 cells during the iron uptake study.	(50)
*in vitro*	ascorbic acid/-	caco-2 cells	While phytic acid, sodium oxalate, and sodium silicate decrease iron absorption, ascorbic acid has the ability to counteract their inhibitory effects.	(51)
*in vivo*	animal tissue/25 g	8 healthy infants 43–49 weeks of age	Inclusion of meat to the vegetable puree significantly increased the nonheme iron absorption.	(64)
*in vivo*	animal tissue/25, 50, or 75 g	45 healthy women with a mean age of 24 ± 3 years	Dose–response increase was observed when pork meat was added to a high-phytate, low-ascorbic-acid meal.	(65)
			While 25 g of meat did not influence the nonheme iron absorption, inclusion of 50 and 75 g of meat increased the absorption significantly (44% and 57%, respectively).	
*in vivo*	animal tissue/-	randomized crossover trial in 21 young women with low iron stores	Addition of fish to high-phytate bean meal enhanced the iron bioavailability.	(66)
*in vitro*	animal tissue/-	caco-2 cell	Caco-2 cells’ response to nonheme iron from infant rice was significantly increased by bovine coproducts (kidney, lung, and heart).	(67)
			For the kidney, lung, and heart, relative uptake of iron was found to be 207.13%, 171.21%, and 265.28%, respectively.	
*in vivo*	phytate/-	58 men and 60 women, aged 19–58 years	Iron absorption was significantly increased by the removal of phytates in bran.	(74)
			The addition of potassium and magnesium phytates in amounts present in bran showed an inhibition of iron absorption.	
*in vivo*	phytate/seven dose levels from 2 to 250 mg	34 men and 90 women, aged 19–47 years	Inhibitory effect of phytate was dose dependent.	(76)
			Ascorbic acid may reduce the inhibitory impact of phytate.	
*in vivo*	phytate/718 to 1190 mg/d in the high-phyate group and 623 to 385 mg/d in the low phytate group	32 nonanemic females, 18–35 years of age, with suboptimal iron stores	Inhibitory effects of phytate on nonheme iron absorption were lessened by eating a high-phytate diet on a regular basis in young women with low iron status.	(79)
*in vivo*	phytate/-	720 pregnant women	Bioavailability of iron and calcium in the diets of pregnant women was inhibited by phytate intake.	(78)
*in vivo*	phytate/77 ± 11 mg	102 females aged between 20 and 30 years	12 weeks of high-phytate wholegrain bread consumption had no effect on iron status in women at reproductive age.	(80)
*in vivo*	phytate/817 ± 21 mg	14 women aged 19–42 years who were not habitually consuming iron-containing nutritional supplements	A significant effect of phytate content on iron absorption was not found when porridge was fortified with iron in the form of either sodium iron EDTA or ferrous sulfate.	(81)
*in vivo*	polyphenols/from 52 to 396 mg	23 males and 54 females aged 19–40 years	Black tea was more inhibitory than cocoa and more inhibitory than herbal teas camomile, vervain, lime flower, and pennyroyal but equivalent to peppermint tea at the same total polyphenol content.	(82)
*in vivo*	polyphenols/20, 50, and 200 mg	97 apparently nonpregnant, nonlactating women aged between 18 and 45 years and weighing below 60 kg	Red bean polyphenols inhibited iron bioavailability dose-dependently. While 20 mg of bean polyphenols had no effect on iron absorption, 50 mg and 200 mg lowered iron bioavailability by 18% and 45%, respectively.	(83)
*in vitro*	polyphenols/-	caco-2 cell	Polyphenolic compounds inhibited the heme iron absorption in a dose-dependent manner.	(121)
			In small amounts of polyphenols (≤4.6 mg/L) ascorbic acid counteracted the inhibitory effect; however, in higher levels (46 mg/L), it could not modulate the inhibition.	
*in vivo*	polyphenols/-	17 mother–child pairs	Polyphenol-rich tea reduced iron absorption from wheat bread fortified with ferrous sulfate or ferrous fumarate by 56–72%.	(89)
*in vivo*	polyphenols/492 mg	50 women aged 21–30 years	In both IDA and nonanemic women, tea consumption decreased iron absorption from NaFeEDTA by more than 85%.	(90)
*in vitro*	polyphenols/-	caco-2 cell	Catechin, 3,4-dihydroxybenzoic acid, kaempferol, and kaempferol 3-glucoside promoted iron uptake, while myricetin, myricetin 3-glucoside, quercetin, and quercetin 3-glucoside showed an inhibitory effect.	(84)
*in vivo*	calcium/100 and 200 mg	788 children aged 6–11 years	As the ascorbic acid and calcium did not exist, iron absorption from the casein/whey-based drink was 20% lower in iron-repleted children than the ones with IDA.	(97)
			Calcium addition decreased the mean iron absorption by 18–27%.	
*in vivo*	calcium/500 mg	13 premenopausal women with pre-existing marginal Fe status aged 28–35 years	Iron absorption from a single meal was reduced from 10.2% to 4.8%.	(95)
			The extent of the calcium impact differed significantly across subjects having similar iron stores.	
*in vitro*	proteins/30 g	simulated gastrointestinal digestion	Iron absorption decreased by the substitution of casein or whey protein for egg white. Mean absorption values fell from 6.67 to 3.65% and 2.53 to 0.98%, respectively.	(122)
*in vivo*	proteins/30 g	15 men and 19 women ranging in age from 18 to 45 years	Iron absorption of completely dephytinized glycinin was found to be 124% compared to egg white; however, relative absorption of completely dephytinized conglycinin was only 44%. Conglycinin fraction of soybean proteins was reported to be an inhibitor of iron absorption.	(105)
*in vivo*	prebiotic/4% of the diet	40 female albino rats (ten-week-old)	Yogurt containing long-chain inulin was more effective for iron absorption than yogurt containing short-chain inulin.	(111)
			Fe_2_(SO_4_)_3_ and long-chain inulin fortified yogurt increased the iron bioavailability. In addition, liver function and the antioxidant capacity were improved.	
*in vivo*	prebiotic/-	24 healthy women aged 35–45 years	No significant differences were observed in heme and nonheme iron bioavailability in the control group.	(109)
			Bioavailability of heme iron from the prebiotic group increased significantly by 56% after prebiotic intake.	
			No significant differences were observed in nonheme iron bioavailability.	
*in vivo*	prebiotic/∼20 g	36 nonpregnant, nonlactating women with low iron status, aged between 18 and 40 years and with a body weight <65 kg	Inulin enhanced the iron absorption by 14% which was statistically insignificant.	(110)

The presence of ascorbic acid in the diet increases the absorption of nonheme iron.^47^ Ascorbic acid aids iron absorption by creating a chelate with ferric iron Fe^3+^ at a stomach acid pH that stays soluble at the alkaline pH of the duodenum, the first section of the small intestine.^48^ In addition, ascorbate, ascorbic acid salt, donates an electron, acting as a free radical scavenger and reducing iron oxidation states to Fe^2+^, which is the only bioavailable form for enterocyte cells.^49^ Fe^2+^ is the only iron that can be absorbed through iron transporters of intestinal enterocyte cells.^11^ To date, numerous *in vivo* and *in vitro* cell culture studies on the effect of ascorbic acid on iron absorption have been reported. Recently, Khoja et al. reported that when ascorbic acid was added into the digesta of plant products (fenugreek sprouts, fenugreek seeds, baobab fruit pulp, and moringa leaves) ascorbic acid enhanced iron absorption in the Caco-2 cell culture model.^50^ He et al. used an *in vitro* digestion/Caco-2 cell model to investigate the effects of phytic acid, sodium oxalate, and sodium silicate on nonheme iron bioavailability in the presence and absence of ascorbic acid. The findings revealed that phytic acid, sodium oxalate, and sodium silicate restrict ferrous iron absorption, but ascorbic acid can counteract this inhibitory impact and enhance ferrous iron uptake.^51^ Similar results were obtained by Villano et al.^52^ who showed that ascorbic acid can resist to the inhibitory effect of polyphenols.

The effect of vitamin C has been proven to be dose dependent^53,54^ and can increase the absorption of iron only when both nutrients are consumed together.^55^ It has been reported that iron absorption gradually increases from 0.8% to 7.1% when increasing amounts of ascorbic acid, ranging from 25 to 1000 mg, are added to a liquid formula meal containing 4.1 mg of nonheme iron. Moreover, it also has been reported that although 500 mg of ascorbic acid taken with food increases the absorption of iron six times ascorbic acid taken 4–8 h before is less effective.^56^ In this sense, incorporation of ascorbic acid into the diet may seem effective for higher intake of iron; however, besides the technical difficulties during the preparation and storage due to the instability of ascorbic acid,^47^ studies have proven that the addition of ascorbic acid to the whole diet has a more negligible effect on increasing iron absorption.^57−60^ In this sense, the impact of meal composition and the whole food matrix in the diet may influence ascorbic acid and may explain the dramatic improvements in iron absorption.

Animal tissues, such as beef, chicken, fish, pork, and lamb, have a positive effect on dietary nonheme iron absorption.^61^ The enhancing effect of animal tissues on nonheme iron absorption was first reported by Layrisse et al. Veal muscle, veal liver, and fish were demonstrated to enhance nonheme iron absorption by 150% in human subjects consuming meals of maize and black beans.^62^ Later, Bjorn-Rasmussen and Hallberg reported that adding chicken, beef, fish, or calf thymus to a maize meal enhanced nonheme iron absorption. As a result of the study, it was concluded that meat increases absorption by inactivating the luminal factors that prevent iron absorption. The authors stated that the most likely mechanism for this effect is the formation of a luminal transporter that transports iron to the mucosal cell membrane.^63^ Engelmann et al. added 25 g of lean beef to the 80 g of vegetable puree, and as a result, an increase in absorption of nonheme iron in infants was observed.^64^ Bech et al. added pork meat to a meal presumed to have low iron bioavailability. In consequence, they observed that the addition of a small amount of pork (>50 g) to a meal that has high inhibitory and low enhancer components (7.4 mg of vitamin C and 220 mg of phytate) increases the bioavailability of iron in a dose-dependent manner.^65^ Navas-Carretero et al. studied the effect of consuming fish (salmon) on nonheme iron bioavailability from a phytate-rich bean meal. They reported that adding fish to the bean meal significantly enhanced iron absorption in iron-deficient women.^66^ A more recent study by O’Flaherty et al. showed that fortification of kidney, heart, and lung meats into infant rice cereals improved nonheme iron absorption to 207.13%, 265.28%, and 171.21%, respectively.^67^ The mechanism of enhancing the effect of animal tissues known as the meat factor has not been identified.^68^ Hurrell et al. attempted to classify the meat factor in this sense. Nonheme iron absorption was improved by 180% and 100%, respectively, when freeze-dried beef and chicken muscle were compared to egg albumin. Muscle tissue has been reported to have a protein- and/or peptide-related influence on iron absorption. However, it was also reported that other variables such as glycosaminoglycans might have played a role as well.^69^

Phytate and polyphenols are the major iron absorption inhibitors in plant-based foods because they make a complex with dietary iron in the gastrointestinal tract.^9^ Phytate is a naturally occurring component found in plants, and it has an inhibitory effect on the bioavailability of most minerals.^70,71^ Phytate cannot be digested by the human body and cannot be absorbed in the small intestine due to the lack of endophytases.^72^ As a result, minerals chelated in phytic acid are not bioavailable.^73^ Hallberg et al. removed phytates in bran by endogenous phytase to observe how its removal impacts absorption and reported that iron absorption is significantly increased in the absence of phytate.^74^ Troesch et al. reviewed the evidence from human studies investigating the impact of phytase on iron and zinc bioavailability. They concluded that phytase promotes the absorption of iron and zinc from phytate-rich meals and can potentially improve magnesium, calcium, and phosphorus absorption.^75^ The dose-dependent inhibitory impact of sodium phytate on iron absorption was investigated by Hallberg et al. Wheat rolls with no phytates and including seven dose levels from 2 and 250 mg were served to humans. It was reported that phytate’s inhibitory impact was significantly dependent on the phytate amount. While 2 mg inhibited the iron absorption by 18%, this value reached up to 82% by 250 mg. Moreover, the impact of ascorbic acid was also evaluated in the study, and the addition of ascorbic acid was observed to counteract the inhibitory effect of phytates.^76^

The inhibitory effect of phytate is considered to be exaggerated for iron in single test meal studies compared with that achieved in whole diets.^77^ For instance, in a single meal study by Al Hasan et al., it has been reported that phytate intake inhibits the bioavailability of iron and calcium from the diets of pregnant women.^78^ On the contrary, Armah et al. demonstrated that eating a high-phytate diet regularly can lessen the inhibitory effects of phytate on nonheme-iron absorption in young women with low iron status.^79^ Similarly, Hoppe et al. reported that consuming low-phytate whole grain bread on a whole diet had no significant effect on iron status compared to consuming high-phytate wholegrain bread.^80^ Previously, Mendoza et al. investigated iron absorption from porridges prepared from genetically modified strains of low-phytate maize and unmodified wild-type maize, both of which were fortified with either ferrous sulfate or sodium iron EDTA. They revealed no significant impacts of modified low phytate on improving iron absorption.^81^

Polyphenols are found in the human diet mainly due to their presence in vegetables, cereals, spices, tea, coffee, red wine, and cocoa.^82,83^ Polyphenols are known iron bioavailability inhibitors and are assumed to work similarly to phytate by forming a complex with iron.^84^ The inhibitory impact of polyphenols on iron absorption has been reported in numerous studies.^82,85−87^ Petry et al. investigated the effect of bean polyphenols on iron absorption in humans. To determine the effect of bean polyphenols on iron absorption in the absence of phytic acid, increasing quantities of red bean hulls were introduced as a source of bean polyphenols to noninhibitory bread meals where phytic acid had been destroyed during dough fermentation. Twenty mg of bean polyphenols did not affect iron absorption. In comparison, 50 mg and 200 mg of bean polyphenols lowered iron bioavailability by 18% and 45%, respectively, demonstrating that red bean polyphenols inhibited iron bioavailability in a dose-dependent manner.^83^ Polyphenols have even been reported to inhibit heme iron absorption depending on the dose. It has been proven that ascorbic acid counteracts the inhibitory effect at low polyphenol doses. However, it cannot show this effect at high doses.^88^ A recent study by Ndiaye et al. showed that polyphenol-rich tea reduced iron absorption from wheat bread fortified with ferrous sulfate or ferrous fumarate by 56–72% in Senegalese mother-child pairs.^89^ Lazrak et al. studied the bioavailability of iron from NaFeEDTA when added to wheat flour-based meals in both nonanemic women and women with IDA when consumed with and without traditional Moroccan green tea, which had 492 mg GA/ml polyphenols per meal portion serving size/day. The results showed that tea consumption reduced iron absorption from NaFeEDTA by more than 85% in both IDA and nonanemic women.^90^ The precise mechanism by which polyphenols reduce the bioavailability of nonheme iron is not fully understood.^91^ Interestingly, using Caco-2 cells demonstrated that some polyphenols such as catechin and kaempferol promote iron absorption. In addition, these studies have reported myricetin, myricetin 3-glucoside, quercetin, and quercetin 3-glucosides as inhibitors.^84,92^

Calcium is also known to inhibit iron absorption, but it differentiates from other inhibitors since it affects nonheme iron and heme iron.^93^ The studies suggested that calcium influenced iron absorption by regulating enterocyte iron transporter proteins.^8^ The action of the mechanism has not been fully clarified yet; however, studies trying to ascertain the mechanism are available in the literature. Previously, it has been suggested that calcium inhibition may occur in the final steps of mucosal cell-to-plasma transport after the two forms of iron had entered a common cellular iron pool, possibly because these two forms of iron have different apical mucosal receptors.^93^ However, an opposing view has argued that although there are differences in the apical receptors for heme and nonheme iron, calcium inhibition may occur during the initial entry of iron into the mucosal cell via inhibition of iron transport.^94^ Although the mechanism has not been fully understood, the inhibition effect of calcium on iron bioavailability has been proved. Benkhedda et al. demonstrated that calcium reduced iron absorption from a single meal from 10.2% to 4.8%; however, the calcium impact differed significantly across subjects having similar iron stores. Authors concluded that aside from the levels of iron stored in the body and the kind of diet, physiologic or genetic variables significantly impact iron absorption in people with similar body iron stores.^95^ In this context, factors other than iron status may influence the expression of iron transporters which are responsible for intestinal iron absorption and their cellular location.^8^ Consumption of an iron-fortified milk product that supplied 100% of the required daily iron intake did not improve iron status in iron-deficient women over four months in a randomized controlled study. The results revealed that consumption of iron-fortified milk does not improve the iron status in iron-deficient menstruating women. The authors concluded that the presence of calcium and casein in the product is the reason for the impact.^96^ Walczyk et al. investigated the effect of calcium (0, 100, and 200 mg/test meal) on iron absorption from a casein/whey-based drink fortified with ferrous sulfate in the absence and presence of ascorbic acid (0, 42.5, and 85 mg/test meal) in a series of randomized crossover studies in both iron-replete (IR) schoolchildren and schoolchildren with IDA. Iron absorption from the casein/whey-based drink was 20% lower in IR children than in IDA children without calcium and ascorbic acid. The lowered addition of calcium means iron absorption by 18–27%, with the impact being more significant with higher calcium levels. Calcium salts (used as supplements) and milk/dairy products had similar effects.^97^ Separating calcium and iron intakes would be a solution to this nutritional challenge. However, evidence shows that long-term calcium supplementation taken simultaneously or separately from meals does not affect iron status.^98−101^ These variations in short- and long-term impacts of calcium are most likely due to adaptation to a high calcium intake as an iron absorption regulation mechanism to maintain iron homeostasis^8^

Proteins have been reported to be inhibitors or enhancers of iron absorption depending on their source. While proteins from meat were reported to be enhancers,^63,69,102^ other proteins such as eggs were indicated to be inhibitory.^103^ Kim et al. conducted a comprehensive study comparing the effect of proteins (from pork, egg albumin, egg yolk, soybean, and casein) on iron absorption and intestinal solubility. They reported that adding pork protein enhanced the iron bioavailability and solubility in the intestine, while they were lowest in the presence of egg yolk.^102^ Cook et al. studied the effect of different semipurified proteins, including casein, egg albumen, and isolated soy protein. When egg albumen and casein were replaced in protein equivalent quantities in a semisynthetic meal, close mean absorptions (2.5% and 2.7%) were observed. However, isolated soybean protein decreased the absorption to 0.5%.^104^ Lynch et al. determined the contribution of soybean protein isolate components to the iron absorption inhibitory property of soybean. They concluded that the two main inhibitors of iron absorption in soybean protein isolates were the protein-related part found in the phytic acid and conglycine (7S) fraction.^105^

Dietary fiber is one of the main components of edible plant parts that is resistant to digestion and absorption in the small intestine of humans and undergoes complete or partial fermentation in the large intestine.^106^ Studies investigating the correlation between iron bioavailability and dietary fibers have not demonstrated results that agree with each other.^107^ Insoluble dietary fibers are known to inhibit mineral bioavailability. However, soluble dietary fibers have a smaller effect on intestinal iron absorption.^108^ Weinborn et al. conducted a human study on the effect of a prebiotic mixture composed of soluble fibers (inulin, polidextrose, arabic gum, and guar gum) on heme nonheme iron. They found that while the prebiotic mix improved the heme iron absorption, nonheme iron absorption was unaffected.^109^ Another human study on enhancing the effects of inulin was performed by Petry et al. However, they reported that inulin did not affect iron absorption in adult women.^110^ A recent *in vivo* study on rats was published in 2021 by Mohammed et al. on the bioavailability of yogurt fortified with iron and supplemented by long- and short-chain inulin. Results showed that fortified yogurt with inulin, particularly the long-chain inulin and iron, has a promising impact on treating iron deficiency by enhancing iron absorption.^111^ One possible mechanism for enhancing the impact of prebiotics may be that prebiotics promote iron absorption through prebiotic fermentation by beneficial microorganisms found in the colon producing short-chain fatty acids (SCFAs). SCFAs can contribute to lowering the pH of the luminal content, enhancing iron solubility by increasing the reduction of Fe(III) to Fe(II), and can increase the absorbent surface area by stimulating the proliferation of epithelial cells.^112^

Oxalic acid and oxalates are considered undesirable components in human and animal nutrition. The excessive consumption of plants high in oxalates may cause hyperoxaluria, which might result in kidney and bladder stones and renal edema and calcification in the worst-case scenario.^113^ Oxalic acid was previously reported to inhibit calcium^114,115^ and zinc^116^ absorption. However, its impact on iron absorption is debatable. Rat studies have shown that the effect of adding purified oxalic acid to the diet is neutral.^117,118^ Similarly, a human study reported that the effect of oxalic acid on iron absorption is insignificant. The authors presumed that most of the iron found in the meal is in the ferric form in the gastric and duodenal phases of digestion. When iron is in the ferrous form, such as in ascorbic-acid-rich foods, oxalic acid may limit iron absorption by producing insoluble ferrous oxalate.^119^ On the contrary, Gupta et al. reported oxalic acid as the most significant inhibitory factor for iron and calcium absorption from green leafy vegetables.^120^

In short, nonheme iron absorption is affected by the presence of various nutrients. Polyphenols, phytates, calcium, and specific proteins are known to decrease iron absorption, while ascorbic acid, animal tissues, and some other proteins may improve the absorption. For dietary fibers, discussions are still under debate. Calcium differs from all these nutrients as it decreases the absorption of both nonheme and heme iron. The mechanism of action for this condition has not been fully elucidated. Looking at all these, dietary factors for iron absorption carry crucial importance, especially for individuals experiencing iron deficiency and anemia, which are intensely studied subjects with promising developments.

## 6 Recent Studies on Improving Iron Bioavailability

Various strategies are known and applied to reduce the prevalence of iron deficiency anemia.^123^ In addition, numerous studies have been carried out for years to enhance iron absorption in humans. General estimates of iron bioavailability include *in vitro* studies, animal bioassays, and human trials. *In vitro* methods have been used as an alternative to *in vivo* techniques for estimating mineral bioavailability. Most of the *in vitro* methods have been developed to mimic physiological conditions of the intestine system. Human and animal studies on iron absorption are time consuming and expensive and offer a limited capacity to assess luminal interactions of iron and food ingredients. These factors directed scientists to develop fast and efficient *in vitro* methods that help analyze food–iron interactions and estimate iron bioavailability.

In consequence, a simulation of *in vitro* digestion combined with caco-2 cell monolayers (human epithelial cell line) was developed.^124^ The caco-2 model is considered helpful because it is of human origin, has many characteristics analogous to the intestinal epithelium, takes up nonheme iron, and exhibits uptake properties consistent with *in vivo* observations in humans and animals.^125^ Therefore, this section covers *in vitro* digestion/caco-2 cell culture model studies and *in vivo* human and animal studies. Recent *in vivo* studies to improve iron bioavailability are shown in Table 2 and *in vitro*/caco-2 cell culture model studies in Table 3.

**Table 2:** Recent *in Vivo* Studies on Improving Iron Bioavailability

technique	compound	study system	food	results	reference
encapsulation	iron encapsulated in banana peel matrix	animal bioassay (rat)	tempeh	A significant (*p* < 0.05) increase was observed in serum hemoglobin and iron levels in all groups with the highest value found in an iron matrix dose of 20 ppm.	(137)
	iron and folic acid (FA) bovine serum albumin nanoparticles	animal bioassay (rat)	stirred functional yogurt	Enhancement in the levels of hemoglobin, iron, ferritin, and total protein was observed.	(138)
	microencapsulated liposomal iron pyrophosphate	human trial	iron pyrophosphate sachets	Microencapsulated liposomal iron pyrophosphate sachets showed higher palatability and bioavailability.	(145)
				Serum hemoglobin levels in nonpregnant women of reproductive age were significantly increased.	
	lipoosomal iron	human trial	oral liposomal iron	62% of the patients who completed the treatment responded to oral liposomal iron therapy (mean increases of hemoglobin from 11.4 to 12.6 g/dL).	(146)
				Number of patients with mild iron deficiency was decreased.	
chelation	tripeptide iron complex, ferrous glycinate	animal bioassay (rat)	-	Blood parameters such as hemoglobin, serum ferritin, and transferrin levels as well as growth parameters and mRNA expression which is a marker of iron deficiency showed that the tripeptide iron complex was more efficient than FeSO_4_ or the ferrous glycinate complex in alleviating IDA.	(154)
	desalted duck egg white peptides-ferrous chelate	animal bioassay (rat)	-	In iron-deficient rats, 3 weeks of treatment caused red blood cells, serum ferritin, hemoglobin, and serum iron levels to reach the normal levels.	(152)
				The effects of IDA were reduced more efficiently by desalted duck egg white peptide-ferrous chelate compared to FeSO_4_.	
	whey protein concentrate–iron complex	animal bioassay (rat)	-	In regular weaning and anemic conditions, the WPC–Fe complex supplementation improves iron bioavailability, hemoglobin level, percent apparent digestibility coefficient, and percent retention/intake.	(161)
				In iron-deficient animals, a spray-dried WPC–Fe complex supplementation significantly increased iron digestion and metabolism.	
nanoparticulation	ferric hydroxide-polyphosphate nanoparticles	animal bioassay (rat)	-	Relative iron bioavailability from polyP-FeO NPs was greater by ∼170% relative to FeSO_4_.	(168)
	bio iron(II) nanoparticles	animal bioassay (rat)	yogurt	Bioiron nanoparticles were good sources of bioavailable iron.	(169)
				Bioiron nanoparticles in 200 and 400 μg/mL were safe and enhanced yogurt quality and shelf life.	
	β-lactoglobulin fibril iron nanoparticles	animal bioassay (rat)	-	β-Lactoglobulin fibril iron nanoparticles were digestible and bioavailable without altering the organoleptic features of the food carriers.	(170)
				β-Lactoglobulin fibril nanocomposites showed no toxicity in a rat assay.	

**Table 3:** Recent *in Vitro* Studies on Improving Iron Bioavailability

technique	compound	food	results	reference
encapsulation	iron encapsulated in thermo-resistant modified starch with or without vitamin C	conventionally and sourdough fermented breads	The bioavailability and bioaccessibility of iron from conventially fermented bread were higher in general.	(136)
			Iron transport efficiency represented a wide range (1.16–13.78%).	
			Fortified breads showed bioaccessibility values changing from 41.45 to 99.31%.	
			Type of fermentation affected the degree of iron oxidation during digestion.	
			Iron source, either ferrous sulfate or ferrous lactate, showed an effect on tested parameters but not statistically significant.	
	microencapsulated iron coated by whey protein isolate and a starch-based aqueous coating	tea	Cellular absorption or iron from microcapsules was increased by 73%.	(140)
			Within 30 min of tea brewing, microcapsules reduced the formation of the iron–polyphenol complex in the tea by 60%.	
chelation	iron–casein complex with ascorbic acid	water and milk	Ascorbic acid addition at the molar ratio of 2:1 improved the iron absorption from ICCs and FeSO_4_ to close levels, and absorption levels were significantly higher than ferric pyrophosphate (FePP) with and without ascorbic acid.	(153)
	lentil-derived hydrolyzed protein–iron complex	-	A significant decrease in the anemic condition in caco-2 cells was observed by looking at the mRNA levels of marker genes (divalent metal transporter-1 (DMT1), transferrin receptor (TFR), and ankyrin repeat domain 37 (ANKRD37)) that were induced by iron deficiency anemia.	(157)
	iron–red tilapia viscera hydrolysate complex	-	The highest iron binding ability was obtained by hydrolysate with 42.5% of hydrolyzation degree.	(158)
			4.7 times higher bioavailability compared to free iron salts was obtained in the complex of red tilapia viscera hydrolysate with 42.5% of hydrolyzation degree and iron.	
	whey protein–iron complex	-	Both mineral uptake and ferritin synthesis were better in the case of WP–mineral complexes.	(160)
			Minerals (iron and zinc) complexed with whey protein showed a significantly lower pro-oxidant activity but had higher bioaccessibility (76%) compared to iron salts alone (68%).	
	whey protein–iron FeCl_2_ and FeSO_4_) complex	-	Complexes prepared with low molecular mass peptides and FeCl_2_ enhanced the iron bioavailability by approximately 70% compared to FeSO_4_.	(159)
			Complexes except for those synthesized with low molecular mass peptides (<5 kDa) increased bioaccessibility value to a level higher than 85%.	

Different approaches, including encapsulation and chelation, are applied to improve iron bioavailability.^15^ Encapsulation is a way of entrapping active agents within a carrier medium and is an effective technique for enhancing the delivery of bioactive molecules and living cells into food.^126^ Encapsulation can be made either in nano or micro forms. The main target of this process is to create a thermodynamically and physically strong barrier against environmental conditions, essentially enzymes, pH, oxygen, and water vapor.^127^ Various studies are available in the literature that encapsulate iron in microstructured emulsion particles,^128^ double emulsions,^129−131^ maltodextrin microparticles,^132^ liposomes,^133,134^ and hydrolyzed glucomannan.^135^ Bryszewska et al. conducted a study in which ferrous sulfate or ferrous fumarate was encapsulated with or without vitamin C, and bread was fortified. The study used both *in vitro* bioavailability cell culture systems (human epithelial adenocarcinoma cell line caco-2) and test tube bioaccessibility approaches.

Both bioaccessibility and bioavailability of iron were higher in encapsulated forms. In addition, the type of iron source, ferrous lactate or sulfate, did not show a significant difference in those parameters. The reason for enhancement was that capsules created a barrier between the iron and food matrix, preventing the interaction between bioavailability inhibitors.^136^ Kusuma and Ermamilia generated microbeads from banana peels to encapsulate the iron, and they assessed the bioavailability of iron-fortified food using the microbeads to fortify tempeh, an Indonesian food. The study applied *in vivo* conditions in 35 male Sprague–Dawley rats of age 2 weeks. A standard diet-fed rat (37 mg of elemental iron) or standard diet plus tempeh with elemental iron dose (10 and 20 ppm), encapsulated iron-fortified tempeh with elemental iron dose (10 ppm 20 ppm), and encapsulated iron-fortified tempeh with elemental iron dose + probiotic (10 ppm probiotic and 20 ppm probiotic). After 4 weeks of treatment, a significant (*p* < 0.05) increase was observed in serum hemoglobin and the iron levels in all groups. The hemoglobin and serum iron levels were the highest in iron-fortified tempeh with elemental iron dose + probiotic group (11.01 6 0.15 g/dl and 340.556 1.55 mg/dl, respectively) followed by encapsulated iron-fortified tempeh with an elemental iron dose group (10.71 6 0.15 g/dl and 338.07 6 4.17 mg/dl). The authors indicated that fortification of tempeh with encapsulated iron enhanced the iron status in iron deficiency anemia.^137^ Darwish et al. formed iron and folic acid (FA) in bovine serum albumin nanoparticles as antianemic pharmacological agents that fortify stirred functional yogurt (SFY). They compared these with a plain control and SFY fortified with iron in free forms. Bioavailability, as well as viscosity, oxidative interactions, and microstructural properties of IDA-induced Albino rats were examined. At the end of the 4-week feeding period, Fe + FA nanocapsule-fortified SFY restored hemoglobin (16.53 gdL1), iron (109.25 gdL1), ferritin (33.25 gdL1), and total protein (8.6 gdL1), with considerable competition demonstrated in calcium and zinc absorbance. This repair in blood values was based on the targeted site-specific delivery of nanocapsules to cells in the digestive tract, which increases bioavailability and solubility. Based on the findings, the authors added that iron (Fe) and folic acid (FA) bovine serum albumin nanoparticles (BSA-NPs) could be suggested as an antianemia supplement in a variety of functional food applications.^138^ Filiponi et al. obtained iron–peptide complexes by reacting small whey peptides (<5 kDa) with ferrous sulfate and encapsulating them by using maltodextrin and polydextrose as wall materials. The results revealed that iron bioavailability from caco-2 cells was significantly higher than their free forms.^139^ Leiva Arrieta,^140^ Bryszewska,^141^ and Cian et al.^142^ studied encapsulated iron absorption as *in vitro* bioaccessibility, and they also observed the enhancement impact of encapsulated iron.

Liposomes having high biodegradability and biocompatibility and being less toxic are used as drug carriers. Liposomal iron is prepared by the microencapsulation method by entrapping micronized iron inside the liposomal layer.^143^ It is a relatively new development with high absorption and lower side effects.^144^ In a study by Hussain et al., the efficacy of microencapsulated liposomal iron pyrophosphate on nonpregnant women at reproductive age with iron deficiency anemia was investigated. The study was carried out with 558 women for 12 weeks. It was observed that the hemoglobin levels in those women significantly increased from 8.71 ± 2.24 to 10.47 ± 1.69.^145^ In a more recent study, de Alvarenga Antunes et al. designed a study on patients who had inactive or mildly active inflammatory bowel disease (IBD) and IDA. The patients took oral liposomal iron for 8 weeks. At the end of the 8 weeks, it was found that oral liposomal iron is effective in improving mild iron deficiency anemia in patients with IBD. Additionally, the quality of life of the patients was increased, and a decrease in fatigue was seen in some patients. Moreover, no adverse effects were observed in the patients.^146^ Liposomal iron confers stability to the molecule and the ability to release the content gradually. In addition, the gradual release aids in better absorption.^145^ Since it leads to a more rapid increase in Hb levels, it may be a potential treatment method for IDA.

Emulsions are one of the promising encapsulation and delivery technologies used mainly in the food and pharmaceutical industries, with advantages such as controlled release and chemical stability of encapsulated nutrients.^147^ Two types of emulsions are available for food grades: water in oil and oil in water emulsions and double ones.^126^ In the literature, several recent studies encapsulate iron in emulsions efficiently.^130,131,148,149^ Looking at a bioaccessibility study, Buyukkestelli and El prepared a double (W1/O/W2) emulsion to encapsulate ferric chloride and measured the bioaccessibility. It was observed that the bioaccessibility of iron was higher in emulsions, and it was directly proportional to the W_2_ phase.^129^ The complex of iron and iron-chelating peptides has been proposed as a much better enhancer of iron bioavailability and iron absorption than ionized iron.^150^ Chelation protects minerals from inhibitors and increases bioavailability by 2–3 times. It overcomes encapsulation instability and the unproven safety of nanoparticles. However, although chelated mineral complexes are promising, their cost limits their use.^151^ Recently, iron-chelating modes of peptides are gaining attention, and numerous studies have been done to clarify their capability to improve iron bioavailability.^150^ Li et al. desalted the salted duck egg white and then formed desalted duck egg white peptide (DPs-Fe) and iron chelate to examine the bioavailability of this complex *in vivo*. While a standard diet was applied to the control group, the positive control group was fed with ferrous sulfate (FeSO_4_), and iron-deficient rats were fed with DPs-Fe. After 3 weeks of treatment, hemoglobin (Hb), red blood cells (RBCs), serum iron (SI), and serum ferritin (SF) levels reached the normal levels in iron-deficient rats. In addition, the DPs-Fe had no adverse effects on the healthy state of rats. In conclusion, the authors declared that DPs-Fe is more effective than FeSO_4_ in alleviating the impacts of iron deficiency. Thus, it was added by the authors that it could be a potential iron supplement for iron-deficient people.^152^

Sabatier et al. developed an iron–casein complex (ICC) with the aim of dairy fortification with iron. However, the study’s main objective was to state the ascorbic acid effect on the *in vitro* bioavailability of ICC compared to ferrous sulfate (FeSO_4_) and ferric pyrophosphate (FePP). Typically, in acidic environments, the solubility of ICC and FeSO_4_ was similar. However, only part of the iron dissociated from caseins. The authors declared that this case indicates that ICC is an iron chelate. Ascorbic acid addition in the molar ratio of 2:1 improved the iron absorption from ICCs and FeSO_4_ to close levels. However, the absorption levels were significantly higher than FePP. The absorption levels were translated into relative *in vitro* bioavailability to FeSO_4_ of 36% for FePP and 114 and 104% for the two ICCs. The authors declared that ICCs with better organoleptic characteristics compared to iron salts could be a good alternative for fortifying dairy products.^153^ Xiao et al. studied the impacts of a tripeptide iron complex (REE-Fe) on iron-deficiency anemia rats. Sprague–Dawley rats were randomized into seven groups at random: a control group, an iron-deficiency control group, and iron-deficiency groups treated with ferrous sulfate (FeSO4), ferrous glycinate (Fe-Gly), or REE-Fe at low, medium, or high doses. The rats were given various iron supplements intragastrically once a day for 21 days. As a consequence, by looking at the blood parameters such as hemoglobin, serum ferritin, and transferrin levels, as well as growth parameters and mRNA expression which is a marker of iron deficiency, it was observed that REE-Fe was more efficient than FeSO_4_ or Fe-Gly to alleviate IDA.^154^ Peptide–iron complexes can be absorbed as a whole in the form of soluble chelates and kept in the ferrous state in the digestive system. Chelates increase iron bioavailability by allowing ferrous metals to pass through the apical membrane of the intestinal epithelium.^155^ Therefore, the chelation method can regulate iron bioavailability, as proven in studies.

In the past decades, the hydrolysates obtained from proteolytic digestion of various food sources have gained attention since they can bind metal ions and enhance their stability, solubility, and bioavailability due to their spatial structure and diverse residues with chains that can donate electrons.^156^ Evcan and Gulec formed a lentil-derived hydrolyzed protein–iron complex and observed the bioavailability *in vitro* in an anemic caco-2 cell line. The hydrolyzed protein–iron complex showed a significant decrease in the anemic condition in caco-2 cells by reducing the mRNA levels of marker genes (divalent metal transporter-1 (DMT1), transferrin receptor (TFR), and ankyrin repeat domain 37 (ANKRD37)) that were induced by iron deficiency anemia.^157^ Gómez et al. hydrolyzed red tilapia viscera to obtain protein hydrolysate having iron-chelating activity. The iron content of hydrolysate with 42.5% of hydrolyzation degree (RTVH_B) and its fraction was evaluated. Additionally, iron bioavailability was measured indirectly as ferritin synthesis in a caco-2 cell model. The RTVH-B showed the maximum iron binding ability. In addition, the Fe^2+^–RTVH-B complex showed 4.7 times higher iron bioavailability than free iron salts. The authors suggested a potential application of RTVH-B as dietary supplements to improve iron absorption.^158^

Iron is a susceptible element for forming new complexes that can inhibit or enhance its bioavailability. Whey protein has been discussed to enhance iron absorption depending on the complexation.^159^ Shilpashree et al. prepared whey protein–iron and whey protein–zinc complexes to evaluate their oxidative stability, *in vitro* bioavailability in the caco-2 model, and bioaccessibility. It was observed that iron and zinc complexed with whey protein have significantly lower pro-oxidant activity while having higher bioaccessibility (76%) than iron salts alone (68%). Furthermore, the bioavailability of whey protein-bound minerals in caco-2 cells was reported to be significantly higher as compared to free minerals. In conclusion, the authors additionally indicated that inhibition of iron catalytic activity by complexing with whey protein is possible, and both whey protein iron and zinc complexes have the potential to be used as organic fortificants in several foods.^160^ Caetano-Silva et al. prepared whey protein–iron complexes with different ligands, including whey protein hydrolysate having a higher and lower fraction than 5 kDa and whey protein isolate. FeCl_2_ and FeSO_4_ were used as iron sources. The study was carried out at *in vitro* conditions. The bioavailability was measured indirectly as ferritin synthesis in a caco-2 cell model, and bioaccessibility was evaluated after *in vitro* gastrointestinal digestion. Results indicated that complexes prepared with low molecular mass peptides and FeCl_2_ enhanced the iron bioavailability by approximately 70% compared to FeSO_4_. Moreover, except for those synthesized with low molecular mass peptides (<5 kDa), all complexes increased the bioaccessibility value to a level higher than 85%.^159^ Gandhi et al. conducted a study on rats. They assessed iron bioavailability from a spray-dried whey protein concentrate–iron (WPC–Fe) complex in weaning and anemic rats. Ferrous sulfate heptahydrate was used as an iron source. The normal-diet fed rats were subgrouped into control, WPC, FeSO_4_ (as positive standard), and WPC-Fe. Results showed that supplementation of the WPC–Fe complex increased the bioavailability, hemoglobin level, % apparent digestibility coefficient, and % retention. Furthermore, the WPC–Fe complex enhanced HDL cholesterol, catalyzed activity, and reduced LDL/VLDL and total cholesterol levels. The authors declared that the use of the WPC–Fe complex decreases the anemia prevalence in rats to combat anemia and iron-deficiency-related disorders.^161^ Similar findings have also been obtained from studies conducted on a succinylated sodium caseinate–iron complex (Shilpashree et al.)^162^ and lactose–iron complex (Sharma et al.).^163^

Nanotechnology is emerging as a viable technique for effective iron food fortification and drug delivery for the treatment of anemia, and food technologists and researchers are increasingly active in using it.^164^ Nanoparticles have been previously proven to improve the bioavailability of the material.^165,166^ However, even though these nanosized particles have better bioavailability, the mechanism and function of produced ferritin mimetic nanoparticles are unclear.^15^ Besides their positive effects, oral nanoparticles causing adverse effects are still a concern, especially for their application in the food industry.^167^ Most recently, Li et al. prepared highly negatively charged ferric hydroxide-polyphosphate nanoparticles (PolyP-FeONPs) and investigated the iron bioavailability in rat-polarized human intestinal epithelial caco-2 cells. They observed in rats that the relative iron bioavailability from PolyP-FeONPs was more significant (∼170% relative to FeSO_4_).^168^ El-Saadony et al. formed bio iron(II) nanoparticles by using the *Bacillus subtilis* ML6 supernatant that reduces FeCl_3_ and produces biological ferrous nanoparticles (bio-Fe(II) NPs), and they supplemented yogurt with bio-Fe(II) NPs. They concluded that bio-Fe(II) NPs were good and safe sources of bioavailable iron with improved sensory properties.^169^ Shen et al. developed a novel β-lactoglobulin fibril–iron nanoparticle hybrid material for iron supplementation. Biodegradable amyloid fibril and iron nanoparticles were combined to form a hybrid material. The new iron nanoparticles were stable in foods and beverages and did not cluster together. *In vivo* testing revealed that the novel supplement was easily digestible and bioavailable without altering the organoleptic features of the food carriers. Furthermore, in rat toxicological investigations, iron β-lactoglobulin fibril nanocomposites showed no toxicity.^170^

## 7 Conclusion

Iron is an essential element for human life, and due to its electron exchange features, it takes part in oxygen transport, energy production, DNA, RNA, and protein synthesis. It is involved in the structure of many enzymes and/or is necessary for their function. Sufficient iron intake, especially from meat, poultry, and seafood, is necessary to prevent iron deficiency and anemia since these foods are rich in the bioavailable form of heme iron. In underdeveloped and developing countries, mainly meat and fish products fall into the category of expensive products, and people may have limited access to these products. On the other hand, there are people who avoid the consumption of these products in line with their own sensitivities and preferences. These people may be at higher risk for iron deficiency and anemia. In this context, the importance of studies to increase iron bioavailability is indisputable. Encapsulation, emulsification, chelation, and fortification play an important role in increasing the bioavailability and absorption rate of iron. Commercial iron supplements are available for humans suffering from IDA or wishing to get iron as a supplement. However, because of the free iron-dependent radical generation, some of the commercial iron supplements may produce adverse effects in the gut lumen and mucosal area of the intestine. Therefore, preventing iron deficiency through the widespread consumption of iron supplements requires continuous innovation in products and processes and also requires a high level of public awareness.
